# Dough Functional Properties and Bread Quality of Stone-Milled Refined Flours in Comparison to Traditional HRS Wheat Flours

**DOI:** 10.3390/foods15122046

**Published:** 2026-06-06

**Authors:** Deepa Pradhan, Amrita Ray, Shahidul Islam

**Affiliations:** 1 Department of Plant Sciences, North Dakota State University, Fargo, ND 58108, USA; deepa.pradhan@ndsu.edu; 2 Northern Crops Institute, Fargo, ND 58108, USA; amrita.ray@northerncrops.com

**Keywords:** stone-milled refined flour (SRF), hard red spring wheat, dough rheology, bread quality, starch pasting behavior, gluten functionality

## Abstract

While traditional flours, such as roller-milled refined flour (RRF) and stone-milled whole-wheat flour (SWF), are subject to a trade-off between nutritional value and sensory quality, stone-milled refined flour (SRF) offers a balanced composition. Nevertheless, the functionality and baking performance of dough depend on complex macromolecular interactions beyond composition alone. Using three hard red spring (HRS) wheat varieties, this study compares the protein and starch functionality, dough rheology, and bread quality associated with SRF compared to RRF, SWF, and roller-milled whole-wheat flour (RWF). Stronger gluten formation in SRF compared to RRF was evident from its higher maximum torque (62.83 GPU) and gluten aggregation energy (1681.7 GPU) in the Glutopeak analysis. Using a Rapid Visco Analyser (RVA), lower peak viscosity (1725.25 RVU) and higher pasting temperature (89.4 °C) were observed for SRF. It also exhibited higher water absorption (68.93%) than RRF (65.98%), although their dough stability and mixing tolerance were similar. While RRF produced the highest specific bread volume (6.74 cc/g) and softest crumb (2147.13 mN), SRF achieved an intermediate volume (5.51 cc/g) with a 26.4% improvement over SWF. The correlation analysis results indicated that specific volume is positively associated with gluten aggregation parameters and negatively correlated with crumb firmness, confirming that bread quality is primarily governed by gluten structure. Overall, the use of SRF resulted in balanced dough properties and bread quality, making it a viable, nutritionally enriched alternative for both artisanal and commercial baking.

## 1. Introduction

The physical and chemical properties of wheat flour play crucial roles in determining its functional performance, processing behavior, and nutritional value in baked products. Among the key factors influencing flour characteristics, the milling method stands out as particularly significant, as it affects both particle size and the retention of bran fraction in the flour stream [1]. Stone milling and roller milling are the two predominant techniques, each with distinct mechanical actions and resulting flour qualities. Stone milling, which involves abrasion and shear between granite stones, tends to retain bran and germ fractions, producing a coarser flour with higher levels of health-promoting fiber, ash, and carotenoids [2,3]. Conversely, roller milling uses steel rollers to selectively isolate the endosperm, generating a finer, more uniform flour with consistent baking performance but reduced nutritional content due to the removal of bran and germ [4]. Thus, RRF and SWF have limitations in terms of nutritional value and sensory quality, respectively. In contrast, as a result of a more moderate flour refinement process, SRF provides a more balanced flour composition [5].

Flour refinement—that is, the process through which bran is separated from the starchy endosperm, significantly impacts flour functional properties. Accordingly, any variation in the refinement level influences downstream processing characteristics, including dough rheology and baking performance [6]. For instance, increased bran content is typically associated with higher protein and gluten levels; however, it can also disrupt gluten network formation, thereby compromising the starch–gluten matrix and cell structure development during fermentation [7]. Moreover, bran content influences the particle size distribution and starch damage, which are key determinants of the handling and processing performance of dough [8], and generally increases the water absorption capacity (WAC), thereby influencing dough development and final product quality [9].

The milling method and refinement process result in significant differences in composition between roller-milled refined flour (RRF), stone-milled whole-wheat flour (SWF), and stone-milled refined flour (SRF), which could be further influenced by varietal differences [2,5,8]. Flour compositional differences ultimately influence functional properties relevant to dough formation and baking; for example, particle size variations influence WAC and gluten hydration kinetics, which determine the physical properties of dough [10]. Additionally, starch damage—primarily driven by milling properties—alters enzymatic activity and water-binding capacity, which in turn influence dough viscosity, fermentation dynamics, and crumb structure [9,11].

Although SRF has been reported to retain more nutritional components than RRF [5], its functional properties in processing remain underexplored. Despite significant compositional changes, previous studies have reported that protein quality attributes are more strongly affected by genetics and are not significantly affected by the milling or refinement process [5,12]. Notably, protein quality indicators such as the glutenin-to-gliadin ratio and unextractable polymeric protein (UPP) content are closely linked to the elasticity and strength of dough, which in turn influence loaf volume and crumb structure [11]. Therefore, the increased retention of nutritionally enriched bran fractions in SRF may not result in substantial variations in dough behavior related to protein quality. However, it should be kept in mind that overall dough functionality is governed by a complex interplay of biochemical reactions and structural interactions that occur during mixing and baking.

Thus, this study involved a comprehensive evaluation of HRS wheat, examining the interactive influences of the milling method, flour refinement process, and wheat variety on protein and starch functionality. The objective was to investigate how these factors interact to influence dough rheology and baking performance, with particular emphasis on comparing SRF to conventional flour types (SWF and RRF). While previous investigations have largely focused on soft wheat systems [13], this work extends the understanding to hard red spring (HRS) wheat to evaluate whether SRF can deliver balanced processing performance while retaining enhanced nutritional value. Comprehensive studies examining the combined effects of protein functionality, starch behavior, dough rheology, and baked product quality in SRF are limited. Therefore, this study aims to advance the functional understanding of SRF and provide practical guidance for optimizing flour selection in health-oriented baked products.

## 2. Materials and Methods

### 2.1. Experimental Material and Factors

A completely randomized design (CRD) was employed in a 3 × 2 × 2 factorial arrangement, incorporating the following factors: (1) Wheat Varieties: three Hard Red Spring wheat (HRS) cultivars Bolles, ND Frohberg, and LCS Buster; (2) flour types: refined flour and whole wheat flour; (3) milling methods: stone milling and roller milling. In addition to the experimental treatments, commercially sourced flours were included as reference controls for comparison: SRF (Janie’s Mill, Protein: 14.75% as-is basis), RRF (King Arthur, Protein Content 12.67% as-is basis), SWF (Palaouse, 13.98% as-is basis), RWF (Sunrise Flour Mill, 15.73% as-is basis). Wheat grains were sourced from the Spring Wheat Breeding Program at North Dakota State University (NDSU) and grown at three locations in North Dakota, Langdon, Carrington, and Dickinson during the 2022 and 2023 growing seasons. Grain samples from different environments (location-year combinations) were composited within each variety to minimize environmental variability. Each treatment combination included three biological replicates, with two technical replicates per sample. Prior to milling, all wheat samples were cleaned using a Carter Day Dockage Tester (Carter Day International, Minneapolis, MN, USA) to remove broken kernels, foreign material, and other contaminants, thereby ensuring sample consistency.

### 2.2. Milling and Sample Preparation for Experiment

Before milling, wheat grains were tempered to a moisture content of 16% for roller milling [14] and 14% for stone milling [13] for 24 h tempering period to align with the operational requirements of each system. A tempering level of 16% is standard for roller milling of HRS wheat. In contrast, while whole wheat stone milling is often conducted without tempering at natural grain moisture (10–12%) [15], tempering to 13–15% is recommended for producing refined flour via stone milling to improve milling efficiency and yield [13]. In this study, 14% moisture was selected as a practical compromise to enhance milling performance while avoiding excessive moisture that may clog stone furrows and reduce grinding efficiency [15]. Two types of flour: whole wheat flour and refined flour were produced using single-batch milling within each system to minimize variability. All flour data were standardized to 14% moisture to account for variation in final flour moisture content. Stone milling was performed using a New American 26-inch stone mill (Elmore, Morrisville, VE, USA). The mill was operated at a feed rate of 40 lb/h, with the stones set 0.08 mm apart and rotating at 167 rpm. This process yielded both whole wheat flour and refined flour fractions. The roller milling was conducted using a Bühler laboratory roller mill MLU-202 (Buhler Co., Uzwil, Switzerland) consisting of three break (B1–B3) and three reduction (C1–C3) passages following AACCI Method 26-20 [16]. The feed rate of wheat to the mill was set at 130 g/min. Roll gaps were set at 0.38 mm (1st break) and 0.075 mm (3rd break), and 0.050 mm (1st reduction) and 0.038 mm (3rd reduction), with a feed rate of approximately 130 g/min. This process produced three fractions: flour, bran, and shorts. The bran and shorts were further ground using a Perten 3100 hammer mill (LM 3100, Perten Instrument, PerkinElmer, Stockholm, Sweden). In addition to the experimental flours, four commercially available flours were used as controls for comparison (SRF, RRF, SWF, and RWF). The temperature of the flour during milling was maintained between 38 and 40 °C.

### 2.3. Protein Functional Properties

Protein functionality, particularly aggregation behavior, plays a critical role in determining the structural integrity and textural quality of dough and baked products. To assess these properties, the GlutoPeak tester (C.W. Brabender Inc., South Hackensack, NJ, USA) was used to evaluate the kinetics of gluten aggregation under high-shear mixing conditions. This test provides valuable insights into the viscoelastic properties and hydration potential of gluten, which are essential for understanding dough development and end-use performance [17]. The GlutoPeak test is significantly faster and less labor-intensive and requires a much smaller sample size (3–10 g). While traditional methods remain reliable, they are time-consuming and involve more complex handling. In contrast, GlutoPeak provides rapid results (within 1–10 min) and directly measures gluten aggregation behavior through torque resistance under high-shear mixing, making it particularly suitable for efficient evaluation of wheat gluten quality [18]. In this procedure, flour samples equivalent to 8 g at 14% moisture basis were mixed with 10 mL of a calcium chloride (0.5 M CaCl_2_) solution in a stainless-steel sample cup. The addition of CaCl_2_ was used to standardize ionic strength and promote gluten aggregation through calcium-mediated protein interactions, thereby improving measurement consistency. The test was conducted at a constant paddle rotation speed of 2700 rpm, with the sample cup temperature maintained at 34 °C using a water-jacketed cooling system. All measurements were performed in duplicate to ensure reproducibility.

### 2.4. Starch Functional Properties

Starch pasting properties serve as key indicators of textural behavior and processing performance in flour-based food systems. These properties reflect the structural transitions during gelatinization, including granule swelling and amylose leaching and retrogradation, involving the reassociation of amylose and amylopectin chains which collectively influence viscosity, stability, and shelf life. To quantify these behaviors, the pasting profile of flour samples was evaluated using a Rapid Visco Analyzer (RVA 4500, Perten Instruments, Macquarie Park, New South Wales, Australia), following the AACCI Approved Method 76.21.02 [16]. Flour sample (3.0 g, adjusted to 14% moisture basis) was mixed with 25.0 mL of distilled water in an RVA canister. The slurry was equilibrated at 50 °C and stirred at 960 rpm for the first 10 s to ensure uniform dispersion, followed by 160 rpm for the remainder of the test. The temperature profile consisted of holding at 50 °C for 1 min, heating to 95 °C over 3 min 42 s, holding at 95 °C for 2 min 30 s, cooling to 50 °C over 3 min 48 s, and a final hold at 50 °C, with a total run time of 13 min. Viscosity data were recorded at 4 s intervals and provides critical parameters including peak viscosity, breakdown, setback, and pasting temperature, offering insight into flour performance in applications such as thickening, gelling, and baking.

### 2.5. Dough Physical Properties

The physical properties of dough were evaluated using a farinograph (Farinograph-TS; Brabender GmbH & Co. KG, Duisburg, Germany), a standard instrument in cereal science for assessing flour mixing behavior and hydration capacity following AACCI Method 54-21.02 [16]. All measurements were conducted on a 14% moisture basis. Water absorption was determined as the amount of water required to achieve a dough consistency of 500 Brabender Units (BU). Key parameters measured included WAC, dough development time (DDT), dough stability (DS), and mixing tolerance index (MTI). These metrics provide insight into the strength, consistency, and tolerance of dough during mechanical handling.

### 2.6. Bread Preparation

Bread samples were prepared from both whole wheat and refined flours produced through stone and roller milling. The baking process followed the straight dough method as outlined in AACCI Method 10-09.01 [16], with minor modifications, including the use of fungal amylase (SKB 15) in place of malt dry powder, instant dry yeast (1%) instead of compressed yeast, the addition of ammonium phosphate (5–10 ppm), and incorporation of 2% shortening. For each flour type, ingredients included 100 g of flour, yeast (1 g/1%), sugar (5%) and salt (1%) incorporated via salt and sugar solution prepared in water, shortening (2.2 mL), α-amylase (1 mL), ammonium phosphate (1 mL), ascorbic acid (1 mL), and a portion of the water were mixed for 3–5 min to form a uniform dough. The dough was then fermented for 95 min at 88 °F (31 °C), followed by a punching, a second proofing phase of 25 min at the same temperature and baking in an oven at 425 °F/218 °C for 25 min. After baking, loaves were cooled, and loaf volume was determined using the rapeseed displacement method following AACCI Method 10-05.01 [16]. Loaf weight was recorded, and specific volume (cc^3^/g) was calculated by dividing volume by weight. Crumb firmness was assessed using a TA-XT2 Texture Analyzer (Texture Technologies Corp., Hamilton, MA, USA), following AACCI Method 74-09.01 [16].

Each bread type was prepared with two biological replication, and manual scoring of loaves was conducted according to AACCI Method 10-12.01 [16], evaluating loaf symmetry (uniformity and shape), crust color (intensity and uniformity of browning), and overall appearance (external defects, surface smoothness, and visual acceptability) based on the standardized scoring scale defined in the method. All evaluations were performed by same assessor to ensure consistency across samples. Bread slices were further analyzed using C-Cell imaging (Calibre Control International Ltd., 5-6 Asher Court, Lyncastle Way, Appleton Thorn Trading Estate, Warrington, UK) to quantify internal crumb structure characteristics, including slice brightness, color, number of cells, cell volume, and uniformity, based on AACCI Method 10-18.01 [16] with slight modification.

### 2.7. Scanning Electron Microscopy (SEM) Imaging of Bread

Bread crumb samples were prepared for SEM analysis to examine the internal microstructure. SEM analysis included three technical replicates per treatment, captured from multiple regions of each sample at different magnifications. Representative images are presented to illustrate the observed structural characteristics. Small sections of the crumb were affixed to cylindrical aluminum stubs using adhesive carbon tabs. Loose particles were gently removed using a nitrogen gas stream to prevent surface contamination. The samples were then coated with a thin conductive layer of gold using a Cressington 108 auto sputter coater (Ted Pella Inc., Redding, CA, USA) to minimize charging during imaging. Micrographs were captured using a JEOL JSM-6490LV scanning electron microscope (JEOL USA, Peabody, MA, USA) at an accelerating voltage of 15 kV.

### 2.8. Statistical Analysis

The experiment included three biological replications for all parameters except baking trials, which included two biological replications due to resource limitations. For each biological replicate, analyses were conducted in duplicate (technical replicates), and the mean of technical replicates was used for statistical analysis. Data were first recorded and organized using Microsoft Excel and then analyzed using R software (version 4.2.2). Analysis of variance (ANOVA) was performed at a 95% confidence level (α = 0.05), and treatment means were separated using Tukey’s Honest Significant Difference (HSD) test. The “Agricolae” and “Multcomp” packages in R were used for post hoc analyses and multiple comparisons. Pearson Correlation analysis was also performed in R (4.2.2) using R packages (correlation, corrplot, and ggplot2) to visualize interrelationships among key traits. Starch damage values were obtained from our previously published study [5] conducted on the same flour samples and were used here solely for correlation analysis with RVA parameters. The data were presented as mean ± standard error, with all assumptions met for valid statistical interpretation.

## 3. Results and Discussion

### 3.1. GlutoPeak Analysis of Gluten Strength During Processing

GlutoPeak is a quick shear-based technique used to evaluate the aggregation behavior of gluten, providing insights into the processing quality of wheat flour during dough mixing [17]. During the test, the gluten proteins in the flour interact to form a cohesive network that resists the motion of the mixing paddle. This resistance is recorded as torque and represented graphically through a torque-versus-time curve, which reflects the strength and stability of this network and is used to predict baking quality parameters such as loaf volume and dough strength [18]. Peak Maximum Time (PMT), aggregation energy (AE), and Torque Maximum (TM) are the most critical GlutoPeak parameters, which directly measure the development speed, overall energy input, and strength of the gluten network, respectively, providing a reliable assessment of dough formation and stability. Other GlutoPeak parameters are generally less consistent and more sensitive to minor variations, making them less informative for directly evaluating flour performance in practical dough applications [19]; furthermore, these additional parameters showed no significant differences between flour types in this study.

While significant differences were observed in PMT and AE across wheat varieties, milling methods, flour types, and their interactions, TM showed no significant differences for flour types (Table S1). The variety Bolles exhibited the strongest gluten performance (highest TM and AE), whereas LCS Buster and the commercial control showed weaker profiles. Stone-milled flours demonstrated faster gluten development (shorter PMT) than roller-milled flours, likely due to increased starch damage enhancing hydration and aggregation. However, the slightly lower TM measured with stone-milled flours suggests reduced peak strength, potentially related to particle size and starch damage differences [5].

Flour type significantly influenced gluten behavior. Refined flours (SRF and RRF) showed higher AE than whole-wheat flours—likely due to the absence of bran [20], which disrupts gluten development and competes for water [21]. However, TM was unaffected, indicating that bran influences gluten development kinetics rather than peak resistance. Table 1 illustrates the interaction between milling method and flour type on gluten aggregation traits. PMT, which reflects the time required for gluten network formation, was the longest in RRF (113.83 s), indicating slower but potentially more consistent gluten development. In contrast, SWF and RWF had shorter PMTs (58–65 s), likely due to rapid yet less-stable aggregation. PMT was highly correlated with SDS-SRC values [5,22], supporting its utility as an indicator of gluten strength. Typically, flours with higher gluten strength exhibit longer PMTs due to slower but more organized aggregation, while weaker flours form gluten networks more quickly but with less cohesion [5,23]. In contrast, SRF showed a moderate PMT (76.17 s) but higher AE (1681.67 GPU), reflecting a well-balanced and extensible gluten network, as is common in an elastic dough. These results are consistent with studies linking longer PMTs and high AE values to stable gluten networks in high-gluten flours [23]. Notably, SRF also recorded the highest TM, indicating a robust gluten structure capable of withstanding higher shear force—an advantageous trait for bread-making applications that require strong dough stability.

Overall, these findings highlight the complex interplay between wheat genotype, milling technique, and flour type in shaping gluten aggregation behavior. SRF stands out due to its combination of moderate PMT, high AE, and high TM, supporting its potential to produce functionally strong doughs with adequate elasticity, rendering it suitable for high-quality baking applications [24].

### 3.2. Starch Pasting Properties by Rapid Visco Analyzer

When a wheat flour–water suspension is heated, its viscosity increases due to starch pasting, as granules absorb water, swell, and gelatinize. This behavior can significantly affect both processing characteristics and the quality of the final product [25]. Therefore, understanding the pasting properties of wheat flour is essential for optimizing dough handling, which ultimately influences the texture of the final product. Key pasting parameters, such as peak viscosity, pasting temperature, and breakdown, reflect the swelling, gelatinization, and stability of starch granules. These properties are affected by various factors, including the genetic background, flour composition, and milling conditions [26]. The pasting temperature marks the onset of viscosity development and reflects the rate of starch hydration and granule expansion toward peak viscosity. These properties are critical for optimizing dough handling and the final product texture across flour types and milling methods.

#### 3.2.1. Peak Viscosity

Peak viscosity was found to be significantly influenced by the wheat variety, milling method, flour type, and their interactions (Table S1). The Bolles and ND Frohberg varieties exhibited the highest peak, hot paste, and final viscosities. Roller-milled flours showed higher peak viscosity than stone-milled flours, while the values for whole-wheat flours generally exceeded those of refined flours (Table 2). This trend may reflect greater water interactions from fiber and more intact starch granules in roller-milled and whole-wheat flours [25,26]. In contrast, stone-milled refined flour (SRF) exhibited the lowest peak viscosity (1725.25 RVU), likely due to elevated starch damage and water absorption, which disrupt granule swelling and reduce viscosity [5,23,27]. Conversely, roller-milled refined flour (RRF) showed the highest viscosity—comparable to that of whole-wheat flours—likely due to its uniform particle size and higher proportion of intact starch granules, which promote optimal swelling and gelatinization [26]. Starch damage showed a clear inverse relationship with RVA viscosity parameters, where stone-milled refined flour (SRF) exhibited the highest starch damage (7.72%) and the lowest peak, hot paste, and final viscosities. In contrast, flours with lower starch damage (SWF, RRF, and RWF) displayed significantly higher viscosities, indicating that excessive starch damage disrupts granule integrity and limits swelling capacity during pasting. Additionally, SRF showed the highest pasting temperature, suggesting that increased absorption of water by damaged starch reduces water availability for intact granules, thereby delaying the onset of gelatinization. Overall, these results indicate that while moderate starch damage may enhance hydration, excessive damage negatively impacts the pasting behavior by impairing starch structure and functionality. These findings are consistent with previous studies reporting that increased starch damage reduces peak and final viscosities while elevating the pasting temperature due to disruption of granule integrity and altered water distribution during gelatinization [25,28].

#### 3.2.2. Pasting Temperature

The pasting temperature, which reflects the thermal energy required to initiate starch gelatinization, was significantly affected by variety, flour type, and interactions (Table S1). The Bolles and LCS Buster varieties, as well as the commercial control, exhibited higher pasting temperatures compared to ND Frohberg, suggesting that genotypic differences in starch composition or protein–starch interactions affect the pasting temperature. Refined flours also showed higher pasting temperatures than whole-wheat flours, consistent with prior research indicating that non-starch components such as lipids, proteins, and fibers in bran may interfere with starch gelatinization and lower the pasting temperature [26].

Among all flour types, SRF showed the highest pasting temperature (89.4 °C), while SWF had the lowest (77.6 °C) (Table 2). The elevated pasting temperature of SRF may be associated with higher starch damage, which increases the absorption of water and reduces its availability for intact granules, thereby delaying the onset of gelatinization [29,30]. However, the combined effects of starch damage and flour matrix composition on pasting temperature remain complex and warrant further investigation.

No significant effects were observed for breakdown, peak time, or setback viscosity between milling methods and flour types (Table S1). These parameters are often influenced by the amylose–amylopectin ratio, protein–starch interactions, and flour matrix structure. In this study, the amylose and amylopectin contents, gliadin-to-glutenin ratios, and UPP% were not significantly different across milling methods and flour types, which may explain the uniformity in these starch pasting traits. This outcome aligns with prior studies suggesting that, when starch composition and protein structure remain stable, differences in thermal and retrogradation-related pasting properties are minimal [31,32]. Nonetheless, variability in breakdown and setback could also stem from complex flour matrix interactions that are not fully captured by the dataset used in this study [33,34].

### 3.3. Dough Mixing and Physical Property Analyses

A farinograph was employed in this study to evaluate the physical and mixing characteristics of dough, particularly the WAC, dough development time (DDT), dough stability (DS), and mixing tolerance index (MTI). These parameters are crucial indicators of flour functionality that directly impact dough handling properties and end-product quality. Previous studies have highlighted the utility of the Farinograph in assessing functional dough properties across wheat varieties and flour types [1,35].

#### 3.3.1. Water Absorption Capacity

The flour WAC exhibited significant differences (*p* < 0.001) between varieties, milling methods, and flour types and their interactions, as detailed in Table S2 in the Supplementary Materials. The variety ND Frohberg, stone milling method, and whole-wheat flour (WWF) demonstrated higher WAC compared to their respective counterparts. This aligns with previous studies indicating that wheat variety (genetics), milling method, and flour type significantly influence WAC [1]. Furthermore, the strong interaction effects underscore the complex interplay between the abovementioned factors in determining WAC. The higher WAC in whole-wheat flour, regardless of milling method or variety, is due to the presence of a higher amount of bran particles; in particular, these particles contain pentosans such as arabinoxylans, which have high water-binding properties [36]. The enhanced WAC observed for stone-milled flour combinations can be attributed to the increased starch damage and finer particle size distribution characteristic of this milling process, as reported by Kihlberg et al. [2].

Table 3 shows that SRF demonstrated a significantly higher WAC (68.93%) compared to RRF (65.98%). This increase in WAC can be attributed to multiple factors. For example, stone milling typically leads to higher levels of starch damage due to the intense mechanical shear forces involved; this increases the number of exposed hydroxyl groups on starch granules, thus enhancing their ability to bind water [5]. Furthermore, SRF contains a greater amount of residual hydrophilic components, such as arabinoxylans and dietary fibers [37,38], contributing to its enhanced water-binding capacity. These results suggest that SRF not only potentially improves the nutritional profile through the inclusion of dietary fiber and minerals, but also enhances functional properties (specifically, WAC) which can positively influence overall product quality. From both an economic and quality perspective, increased WAC in flour is advantageous for bread production [39]. Higher water absorption also allows for greater dough yield, which is financially beneficial.

#### 3.3.2. Dough Development Time

A longer dough development time (DDT) often correlates with stronger gluten networks, which provide better dough handling characteristics and improved loaf volume. In this experiment, DDT was found to be significantly influenced by the wheat variety, milling method, flour type, and their interactions. Among the varieties, Bolles (10.35 min) exhibited the longest DDT, while refined flour (8.59 min) exhibited a longer DDT than the whole-wheat flours (Table S2).

The significantly longer DDT exhibited by SRF (9.26 min) compared to RRF and SWF indicates its stronger gluten-forming capabilities. Specifically, SRF showed approximately 17% and 19% increases in DDT over RRF and SWF, respectively (Table 3). Longer development times are typically associated with higher protein content and stronger gluten networks, which are desirable characteristics for bread-making [35]. These results underscore SRF’s superiority in terms of strong gluten network formation and highlight the interplay between varietal traits and milling-induced starch–protein modifications.

#### 3.3.3. Dough Stability (DS)

Higher stability values indicate stronger doughs that can withstand longer mixing times without deterioration of their structure. Dough mixing stability showed significant differences across varieties, milling methods, flour types, and their interactions. In particular, the variety Bolles (33.29 min), roller mill (21.24 min), and refined flour (22.21 min) demonstrated the highest stability (Table S2) among the corresponding levels, indicating a strong gluten network which is capable of withstanding prolonged mixing while maintaining gas retention—a critical factor for artisan breads.

Higher dough stability can be promoted by protein quality and a uniform particle size distribution. Among the varieties, Bolles is characterized by its high protein content (15.09%) and strong glutenin-to-gliadin ratio, which enable the development of a robust gluten network, allowing the dough to withstand prolonged mixing without structural deterioration. Roller milling contributes to stability by producing finer and more uniform particles (<105 µm), which promote consistent hydration and cohesive protein–starch interactions, thus minimizing disruptions during mixing [40]. On the other hand, refined flour lacks bran and germ, which are known to disrupt gluten formation in whole-wheat flours, resulting in a smoother protein matrix and better gas retention [41]. These factors collectively explain the superior dough stability of these combinations, making them ideal for artisan bread applications that require prolonged mixing and strong dough structure. On the other hand, the commercial source (11.40 min), stone mill (17.73 min), and WWF (16.75 min) showed the lowest stability.

Notably, the interaction between milling method and flour type did not significantly influence dough stability (Table 3), which can be attributed to the dominant influence of intrinsic factors such as protein quality and gluten strength that overshadow the combined effects of these factors [42]. Overall, these findings highlight that while variety and protein quality are primary determinants of dough stability, stone-milled refined flour (SRF) shows particularly noteworthy performance, demonstrating a balanced profile with improved stability over whole-wheat flour and enhanced nutritional value compared to roller-milled refined flour. This positions SRF as a promising alternative for baking applications that seek to balance functional performance and nutritional enhancement.

#### 3.3.4. Mixing Tolerance Index

Higher MTI values indicate faster gluten breakdown during mixing, while lower MTI values (<30 BU) correspond to stronger flours with greater resistance to overmixing, making them ideal for bread-making [43]. The MTI is primarily governed by protein quality—specifically, the glutenin-to-gliadin ratio and unextractable polymeric protein (UPP) content—which determine gluten strength and dough elasticity [44]. The MTI was found to be significantly influenced by the wheat variety, milling method, and their interaction (Table S2). The variety Bolles, which is known for its high protein content and strong glutenin-to-gliadin ratio, exhibited a low MTI value (11.33 BU) and high stability (33.29 min), highlighting its suitability for prolonged mixing applications such as those in artisan bread production. In contrast, commercial flour showed the lowest stability (11.40 min) and the highest MTI (21.83 BU), indicating faster gluten breakdown. The lack of variability in mixing tolerance across the milling method and flour type interactions suggests that the resilience of the gluten network depends more on the wheat variety and overall protein composition than on milling-induced changes. This aligns with the findings from SE-HPLC analyses, which showed no significant variations in protein quality profiles in the two-way interaction between milling method and flour type. These results suggest that the milling method and flour type do not substantially alter the intrinsic protein functionality in dough (Table 3).

In summary, the farinograph analysis revealed that SRF exhibited moderate dough stability and MTI, performing better than whole-wheat flour but slightly worse than roller-milled refined flour in terms of mixing strength. While SRF did not match the peak stability of RRF, it maintained desirable functional characteristics with a relatively low MTI, indicating a reasonably strong gluten network. These results underscore SRF’s potential as a functional and nutritionally enhanced alternative to conventional refined flour, particularly for applications requiring a balance between dough strength and extended mixing tolerance.

### 3.4. Baking Properties

End-product quality of different flour types was assessed by baking bread using a straight dough method following AACCI 10-10.03 [16]. Bread quality was evaluated using both manual scoring and advanced imaging techniques, such as the C-Cell system, following the AACCI method. The C-Cell analyzer provides objective, reproducible measurements of crumb structure, including cell size, distribution, and uniformity, making it ideal for assessing internal bread quality [45]. Manual scoring complements this by addressing external characteristics such as loaf shape, crust texture, and esthetic appeal, which are critical for consumer satisfaction. Integrating C-Cell analysis with manual evaluation offers a comprehensive approach to optimizing bread quality in terms of both functionality and sensory attributes.

#### 3.4.1. Bread Specific Volume

Specific volume in bread refers to the volume-to-weight ratio and is a critical quality indicator in end-product quality. Unlike total volume (an extensive property dependent on loaf size), specific volume is an intensive property that standardizes measurements by accounting for mass, enabling direct comparisons between loaves of varying weights while reflecting the loaf’s lightness, aeration, and crumb structure [46]. The specific volume of bread is influenced by various physicochemical properties of the grain and flour. Notably, the content and quality of protein play significant roles in determining a bread’s final volume and texture [47]. These elements, along with other flour properties, work together to shape the bread’s overall appeal.

**Figure 1:**
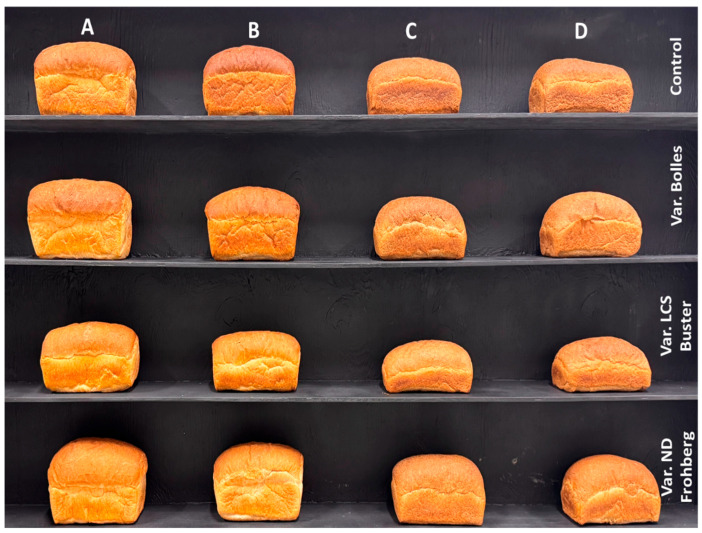
Bread loaf samples made from three varieties and a commercial source (control) with different flour types: (**A**) bread made from RRF, (**B**) bread made from SRF, (**C**) bread made from SWF, and (**D**) bread made from RWF.

The specific volume was found to significantly differ between varieties, milling method, flour type, and interactions between the milling method and flour type (*p* < 0.001) (Table S2). The highest specific volume was observed in the varieties Bolles and ND Frohberg, roller-milled flour, and refined flour compared to their respective counterparts (Figure 1). Particularly, loaves made with RRF (A) exhibited the highest volume across all varieties, followed by SRF (B), which showed only slightly lower volume than RRF. The highest bread specific volume (6.74 cc/g) for RRF (Table 4A) was attributed to its high wet gluten and minimal bran interference, which enhance gas retention and crumb softness, as shown in Figure 1 [48]. A higher specific volume indicates better gas retention during baking, leading to a softer crumb and improved texture.

On the other hand, the lowest specific volumes were observed in the WWF breads (SWF: 4.36 cc/g and RWF: 4.11 cc/g), while the SRF bread showed an intermediate specific volume (5.51 cc/g). This lower specific volume is due to bran and germ disrupting gluten networks and increasing baking absorption (78–79%), limiting the dough’s expansion and leading to a weaker dough structure and reduced gas retention. A similar result has been reported by Roozendaal et al. [49]. However, it should be noted that the refining process increased the specific volume of the SRF breads by 26.4%, when compared to SWF breads.

#### 3.4.2. Baking Water Absorption

Baking absorption indicates the percentage of water required by flour to form an optimal dough consistency at a standardized moisture content (14%). Higher baking absorption typically corresponds to higher fiber content and bran presence, as seen in whole-wheat flours. Baking absorption was found to significantly differ with variety, milling method, flour type, and their interactions (Table S2). In case of the interaction between milling type and flour type (Table 4A), SRF showed higher baking absorption (71.16%) than RRF (68.34%) but was lower than whole-wheat flours (SWF: 79.17%, RWF: 78.32%). This moderate absorption suggests that SRF has a lower bran or fiber content compared to whole-wheat flours but a greater content than roller-refined flour; this suggestion is further justified by its ash content.

#### 3.4.3. Bread Crumb Firmness

Firmness is measured by assessing the force needed to compress or break a sample, serving as an indicator of its softness [50]. Firmness is also a key indicator of the texture, freshness, and staling rate of bread. Softer breads with lower firmness values are often preferred for their fresh and tender crumb, while firmer breads may indicate staling or denser formulations. Bread firmness significantly varied among the varieties, milling method, flour type, and their interactions (Table S2). The variety LCS Buster (7503.88 mN), roller-milled flour (4536.81 mN), and WWF (5345.43 mN) showed the highest firmness values. Table 4A summarizes the influence of milling methods and flour types. A 1.79% decrease in firmness for SRF compared to SWF was observed, indicating that SRF breads have softer crumb texture than SWF breads. RRF breads exhibited the lowest crumb firmness (2147.13 mN), directly influenced by their advanced refining process.

The removal of bran and germ eliminates physical disruptions to the gluten network, while an ultra-fine particle size (typically <105 μm) promotes a homogeneous starch–protein matrix [51]. This uniformity enhances dough elasticity and facilitates uninterrupted starch gelatinization, yielding a softer, more cohesive crumb compared to flours retaining bran (SWF) or balancing partial bran inclusion (SRF). However, this textural superiority comes at a nutritional cost; the absence of bran and germ strips RRF of fiber, antioxidants, and lipids which critical for providing health benefits [44], underscoring the trade-off between sensory quality and nutritional value in refined flour systems.

#### 3.4.4. Bread Quality Assessment by Visual Inspection

Bread physical quality was scored following the AACCI (10-12.01) method [16]. The bread samples were graded in the range of 0–10, with values closer to 10 indicating good quality and values closer to 0 indicating low quality. All parameters of the manual scores showed non-significant differences for the three-way interaction between varieties (Table S3), milling methods, and flour types; however, a significant difference was observed for the interaction between milling method and flour type. Table 4B shows the scoring of bread samples using refined flour bread as a standard. The analysis highlights the significant influences of flour type and milling method on bread quality attributes such as symmetry, crust color, crumb color, and grain texture (*p* < 0.001).

RRF breads achieved the highest scores for symmetry (8.00), crumb color (8.00), and grain texture (7.63), indicating superior bread quality; this is due to its low bran interference and better gluten network formation, which promote a uniform loaf structure, lighter crumb color, and finer grain texture [48]. SRF breads showed moderate scores for symmetry (7.00), crumb color (5.00), and grain texture (5.13). Notably, SRF breads achieved the highest crust color score (7.88), likely due to its higher damaged starch content, which enhances Maillard reactions during baking, producing a visually appealing crust [8]. On the other hand, the whole-wheat flour breads (SWF and RWF) scored the lowest across all parameters due to bran and germ disrupting gluten networks, increasing water absorption, and contributing to darker crumb colors and coarser textures [52]. These results align with earlier findings where refined flours resulted in higher specific volumes and softer crumb structures compared to the whole-wheat flours, further emphasizing their suitability for producing high-quality bread.

#### 3.4.5. Image-Based Physical Quality Evaluation Using C-Cell

C-Cell was used to objectively evaluate crumb characteristics by digitally capturing cross-sectional images of bread slices. Parameters such as brightness, crumb color (L*, a*, b*), number of cells, and cell volume are commonly measured to assess the internal crumb structure and overall quality of bakery products. The C-cell data (Table S4) demonstrated that brightness was significantly different under all main effects (variety, milling method, flour type) and their interactions, except for the three-way interaction. The CIELAB color parameters L* and a* were found to significantly differ for the main effect (milling method and flour type), while b* was found to not significantly differ under all treatments. Table 4C presents the C-Cell data, as influenced by the mill × flour interaction. Bread made from SRF exhibited moderate brightness (78.06), lower than that of RRF bread (114.01) but higher than those of WWF breads (SWF: 65.09; RWF: 59.45). This indicates that the use of SRF produces bread with intermediate crumb brightness, lighter than whole-wheat flours but darker when compared to roller-refined flour. This result aligns with previous findings, with roller milling typically yielding brighter flours due to more efficient removal of bran particles compared to stone milling [53]. Similarly, for the CIELAB color parameters, a higher L* value was observed in RRF bread (48.24), followed by SRF bread (33.52), indicating a more appealing and brighter bread color than that of breads made from WWFs, which produced denser and darker bread due to bran pigments. SRF showed moderate redness values (a* = 3.70), indicating a slightly redder hue compared to RRF (−0.41), likely due to the retention of bran pigments. Yellowness (b*) was statistically similar across all samples, suggesting a minimal impact of milling method or refinement on yellowness.

#### 3.4.6. Crumb Structure and Texture Evaluation Using C-Cell

The C-Cell system’s quantitative analysis of crumb microstructure revealed critical linkages between the milling technique, flour composition, and consumer-perceived texture. The number of cells was found to significantly differ by the milling method, flour type, and their interaction (Table S4) but was not significantly influenced by variety, indicating that the processing method can sometimes overshadow genetic differences in determining product quality [54]. Table 4C summarizes the influence of the milling method and flour type interaction on the crumb structure and texture, as evaluated using C-Cell. RRF breads exhibited the highest cell count (3332.6), reflecting their uninterrupted gluten networks and superior gas retention. Uniform cellular architecture translated to a light, springy crumb, ideal for soft, commercial-style breads. In contrast, stone- and roller-milled whole-wheat flours (SWF/RWF) showed sparse, irregular cells due to bran fragments disrupting gluten development, resulting in a grainy, dense texture, which is often criticized in whole-grain products.

SRF bread showed an 8.70% increase in cell count compared to SWF (2774.88 cells), indicating a significant improvement in bread texture. Its moderately open crumb structure balances fine aeration (smaller, uniform cells) and artisanal heartiness (slightly larger, clustered cells), appealing to consumers seeking nutrient-rich bread without extreme graininess. Notably, while the cell volume remained statistically similar across the interaction between milling method and flour type, C-Cell’s measurements of the number of cells and their uniformity revealed textural details that cannot be noticed through manual evaluation. In fact, C-Cell data can help to validate manual scores by providing quantitative metrics such as the number of cells, cell volume, and crumb color [55].

### 3.5. Correlation Analysis

**Figure 2:**
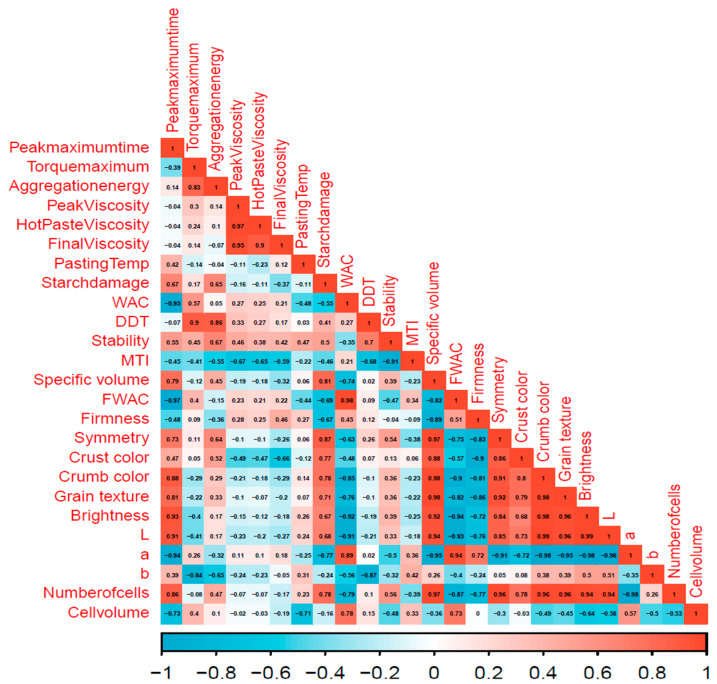
Pearson correlation matrix for functional, rheological, and bread quality parameters in wheat flour systems.

The correlation analysis (Figure 2; corresponding significance levels are provided in Supplementary Materials Figure S1) revealed the relationships among starch damage, hydration properties, pasting behavior, gluten functionality, and bread quality parameters, highlighting the complexity of flour and baking systems. Starch damage was significantly negatively correlated with RVA viscosity parameters (peak, hot paste, and final viscosity), indicating that excessive granule disruption reduced swelling capacity and paste stability, consistent with previous findings [25]. However, its relationship with pasting temperature was weak and non-significant, suggesting that gelatinization behavior is influenced more by the flour matrix composition than starch damage alone. In contrast, starch damage showed a positive association with farinograph water absorption within individual flour systems, reflecting enhanced hydration capacity [56]; however, this relationship became significantly negative when all samples were combined. This shift was driven by compositional differences, as whole-wheat flours exhibited significantly higher water absorption—despite lower starch damage—due to the dominant contributions of arabinoxylans and fiber, which possess greater water-binding capacity than starch [57].

Gluten aggregation parameters (Peak Maximum Time, torque, and aggregation energy) were significantly positively correlated with the farinograph indices (DDT and stability), confirming that gluten network development governs dough strength. These properties were also significantly associated with bread quality, where specific volume showed a strong positive correlation with gluten aggregation and a significant negative correlation with crumb firmness, indicating that loaf expansion was primarily controlled by gluten structure rather than starch pasting properties alone. Additionally, cell structure parameters (cell number and cell volume) were significantly correlated with specific volume, linking microstructure to macroscopic quality attributes [46]. Overall, these results demonstrate that milling-induced changes in starch damage and particle structure affect water distribution and gluten development; however, their impacts on functional and baking performance must be interpreted within the broader context of flour composition, where fiber, protein, and starch interactions collectively determine dough behavior and final product quality.

### 3.6. Microstructure Observations of Bread Samples by Scanning Electron Microscopy (SEM)

**Figure 3:**
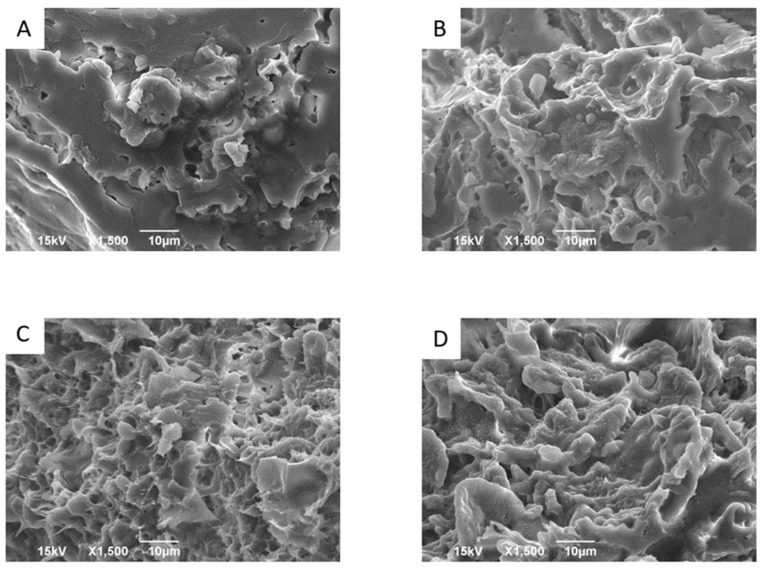
Scanning Electron Microscopy (SEM) images of bread samples (using the Bolles variety) at 15 kV and 1500× magnification: (**A**): roller-milled refined flour (RRF) bread, (**B**): stone-milled refined flour (SRF) bread, (**C**): stone-milled WWF (SWF) bread, and (**D**): roller-milled WWF (RWF) bread.

Scanning electron microscopy (SEM) images of the bread samples (Figure 3) offer qualitative insights into the variations in microstructural composition among the bread samples made from SRF, SWF, RRF, and RWF using the Bolles variety. SEM images obtained at 15 kV and 1500× magnification revealed distinct microstructural differences in the bread samples, focusing on the texture, structure, and quality of the baked products. The RRF bread showed a smooth and cohesive protein–starch matrix with minimal disruptions, indicative of strong gluten development and excellent gas retention as a result of its refined nature, which minimizes bran interference and promotes a uniform crumb structure. This structure also correlates with RRF’s superior dough stability and elasticity, as evidenced by its high Peak Maximum Time (PMT) in the GlutoPeak tests (Table 1). This also aligns with the RRF bread’s highest specific volume (6.74 cc/g) and softer crumb texture in the baking tests (Table 4A).

The SEM analysis also revealed that SRF bread exhibits a distinctive microstructure characterized by pronounced lamellar formations and larger cavities, creating a more open network compared to RRF. This unique structure could be due to milling-induced thermal effects. These microstructural characteristics correlate with the SRF bread’s intermediate specific volume (5.51 cc/g) and moderate crumb firmness (Table 4A), positioning it as a favorable option lying between refined and whole-wheat flours.

In contrast, WWF breads show more irregular arrangements with disrupted starch networks, which may be associated with the presence of bran particles and non-starch components [58]. The SWF bread exhibits particularly large voids and damaged granules that reduce gas retention capacity, explaining its denser crumb structure. The RWF bread shows a heterogeneous distribution of components, with an indication of fiber elements weakening the gluten network [59]. Importantly, the observed microstructural variations were consistent with the trends observed in the instrumental and baking analyses. Samples exhibiting more continuous matrices (e.g., RRF) corresponded to higher dough stability, gluten aggregation parameters, and specific volume, while more heterogeneous structures (e.g., SWF) were associated with lower volume and increased crumb firmness. The SRF sample displayed intermediate structural characteristics, with a partially continuous matrix and moderate pore development, aligning with its intermediate dough strength, specific volume, and crumb firmness relative to RRF and whole-wheat flours. These associations suggest that microstructural organization is linked to functional performance; however, the SEM results should be interpreted as supportive rather than definitive evidence. Similar results were also observed for the other varieties, as shown in Supplementary Materials Figure S2.

## 4. Conclusions

Stone-milled refined flour (SRF) demonstrated balanced performance, occupying an intermediate position between roller-milled refined flour (RRF) and whole-wheat flours (SWF and RWF) in terms of the rheological and baking properties of dough. Although SRF did not achieve the same performance level as RRF, it showed clear improvements over SWF in several key functional and quality attributes. These improvements included enhanced gluten functionality, as evidenced by its higher GlutoPeak aggregation energy (1681.7 vs. 1485.66 GPU); greater dough stability (20.72 vs. 14.73 min); more balanced water absorption (68.93% vs. 74.45%); increased bread volume (5.51 vs. 4.36 cc/g); improved bread symmetry; enhanced crust and crumb color; and improved crust texture, quantified through a higher number of cells (3016.4 vs. 2774.9). The correlation analysis results further revealed that bread volume was positively associated with gluten aggregation parameters, indicating that the improved bread quality of SRF relative to SWF was primarily driven by enhanced gluten functionality. In addition, SEM observations showed that the SRF bread possessed an intermediate crumb microstructure, lying between those of refined and whole-wheat systems. Overall, these findings position SRF as a practical intermediate flour that offers improved nutritional value while maintaining acceptable baking performance. Nevertheless, this study did not explicitly evaluate genotype × environment (G × E) interactions, which may influence the stability of functional and baking traits across different environments. Furthermore, the baking evaluation was limited to bread products; therefore, additional studies are needed to assess the performance of SRF across a broader range of food products and processing conditions.

## Figures and Tables

**Table 1:** Gluten aggregation properties of flour samples, as influenced by the milling process and flour type.

Sample Code	GlutoPeak Analyses		
	Peak Maximum Time (Sec)	Torque Maximum (GPU)	Aggregation Energy (GPU)
SRF	76.17 ± 4.61 ^b^	62.83 ± 1.95 ^a^	1681.7 ± 48.31 ^a^
SWF	65.58 ± 1.84 ^bc^	60.92 ± 2.38 ^a^	1485.66 ± 61.62 ^c^
RRF	113.83 ± 9.47 ^a^	56.17 ± 2.41 ^b^	1580.85 ± 64.94 ^b^
RWF	58.83 ± 1.84 ^c^	59.33 ± 3.52 ^ab^	1475.57 ± 87.62 ^c^

Data represent mean ± standard error. Values with different superscripts in a column differ significantly (*p* < 0.05). SRF: stone-milled refined flour; SWF: stone-milled WWF; RRF: roller-milled refined flour; RWF: roller-milled WWF.

**Table 2:** Pasting properties of flour samples, as influenced by the milling method and flour type.

Sample Code	RVA Analyses				Starch Damage (%)
	Peak Viscosity (RVU)	Hot Paste Viscosity (RVU)	Final Viscosity (RVU)	Pasting Temperature (°C)	
SRF	1725.2 ± 83.17 ^b^	1474.0 ± 90.19 ^b^	2086.4 ± 87.38 ^b^	89.4 ± 2.49 ^a^	7.72 ± 0.47 ^a^
SWF	2212.3 ± 41.51 ^a^	1759.7 ± 36.81 ^a^	2533.0 ± 39.14 ^a^	77.6 ± 2.42 ^c^	5.93 ± 0.09 ^c^
RRF	2196.5 ± 62.18 ^a^	1700.3 ± 40.13 ^a^	2556.7 ± 61.07 ^a^	84.0 ± 3.02 ^b^	6.33 ± 0.12 ^b^
RWF	2144.6 ± 34.88 ^a^	1654.5 ± 37.09 ^a^	2636.2 ± 54.86 ^a^	85.4 ± 2.60 ^ab^	5.28 ± 0.25 ^d^

Data represent mean ± standard error. Values with different superscripts in a column differ significantly (*p* < 0.05). SRF: stone-milled refined flour; SWF: stone-milled WWF; RRF: roller-milled refined flour; RWF: roller-milled WWF.

**Table 3:** Farinograph analysis of flour samples, as influenced by the milling method and flour type.

Sample Code	Farinograph Analyses			
	Water Absorption Capacity (%)	Dough Development Time (min)	Stability (min)	Mixing Tolerance Index (BU)
SRF	68.93 ± 0.98 ^b^	9.26 ± 0.41 ^a^	20.72 ± 2.77 ^a^	16.67 ± 2.58 ^a^
SWF	74.45 ± 0.66 ^a^	7.78 ± 0.51 ^b^	14.73 ± 1.76 ^a^	17.33 ± 1.23 ^a^
RRF	65.98 ± 1.29 ^c^	7.92 ± 0.69 ^b^	23.71 ± 3.21 ^a^	14.00 ± 0.81 ^a^
RWF	73.99 ± 0.84 ^a^	8.35 ± 0.63 ^ab^	18.76 ± 3.29 ^a^	16.17 ± 1.61 ^a^

Data represent mean ± standard error. Values with different superscripts in a column differ significantly (*p* < 0.05). SRF: stone-milled refined flour; SWF: stone-milled WWF; RRF: roller-milled refined flour; RWF: roller-milled WWF.

**Table 4:** (**A**). Bread quality parameters, as influenced by milling method and flour type. (**B**). Scoring of bread samples, as influenced by milling method and flour type. (**C**). Bread quality parameters determined via C-Cell, as influenced by milling method and flour type.

(**A**)						
**Sample code**	**Specific Volume (cc/g)**		**Baking Abs (14%)**		**Firmness (mN)**	
SRF	5.51 ± 0.26 ^b^		71.16 ± 1.21 ^c^		3696.88 ± 728.36 ^b^	
SWF	4.36 ± 0.20 ^c^		79.17 ± 0.72 ^a^		3764.38 ± 800.51 ^b^	
RRF	6.74 ± 0.25 ^a^		68.34 ± 1.48 ^d^		2147.13 ± 288.80 ^c^	
RWF	4.11 ± 0.22 ^c^		78.32 ± 1.10 ^b^		6926.50 ± 1439.79 ^a^	
(**B**)						
**Sample code**	**Symmetry**	**Crust Color**	**Crumb Color**		**Grain Texture**	
SRF	7.00 ± 0.38 ^b^	7.88 ± 0.23 ^a^	5.00 ± 0.27 ^b^		5.13 ± 0.4 ^b^	
SWF	6.00 ± 0.27 ^c^	5.88 ± 0.23 ^c^	3.13 ± 0.13 ^c^		4.75 ± 0.31 ^b^	
RRF	8.00 ± 0.33 ^a^	7.25 ± 0.37 ^b^	8.00 ± 0.27 ^a^		7.63 ± 0.46 ^a^	
RWF	5.88 ± 0.23 ^c^	6.13 ± 0.3 ^c^	2.75 ± 0.16 ^c^		4.00 ± 0.33 ^b^	
(**C**)						
**Sample code**	**Brightness**	**Crumb Color via C-Cell**			**Number of Cells**	**Cell Volume (mm^3^)**
		L*	a*	b*		
SRF	78.06 ± 1.52 ^b^	33.52 ± 1.57 ^b^	3.70 ± 0.16 ^b^	14.54 ± 2.1 ^a^	3016.4 ± 48.44 ^b^	32.80 ± 2.20 ^a^
SWF	65.09 ± 3.11 ^c^	24.8 ± 1.01 ^c^	6.06 ± 0.15 ^a^	16.23 ± 0.66 ^a^	2774.9 ± 44.26 ^bc^	34.63 ± 3.38 ^a^
RRF	114.01 ± 2.35 ^a^	48.24 ± 2.21 ^a^	−0.41 ± 0.31 ^c^	17.71 ± 1.78 ^a^	3332.6 ± 90.65 ^a^	31.38 ± 0.89 ^a^
RWF	59.45 ± 3.30 ^c^	24.29 ± 0.89 ^c^	5.64 ± 0.26 ^a^	14.54 ± 0.54 ^a^	2741.5 ± 69.13 ^c^	37.88 ± 5.77 ^a^

Data represent mean ± standard error. Values with different superscripts in a column differ significantly (*p* < 0.05). SRF: stone-milled refined flour; SWF: stone-milled WWF; RRF: roller-milled refined flour; RWF: roller-milled WWF.

## Data Availability

Data will be made available on request.

## Supplementary Materials

The following supporting information can be downloaded at: https://www.mdpi.com/article/10.3390/foods15122046/s1; Table S1. GlutoPeak and RVA analyses of flour samples (Mean Values); Table S2. Farinograph analyses and baking quality of flour samples (Mean Values); Table S3. Visual Inspection of bread samples; Table S4. C-Cell analysis of bread samples; Figure S1. Pearson correlation matrix for functional, rheological, and bread quality parameters in wheat flour systems. Significance levels are indicated as *p* < 0.05 (*), *p* < 0.01 (**), and *p* < 0.001 (***); Figure S2. Scanning Electron Microscopy (SEM) images of bread samples using flour derived from the ND Frohberg (Top 4) and LCS Buster (Bottom 4) varieties at 15 kV and 1500× magnification.
