# Supplementary material for: Dough Functional Properties and Bread Quality of Stone-Milled Refined Flours in Comparison to Traditional HRS Wheat Flours
Source: Foods. 2026 Jun 6;15(12):2046. doi: 10.3390/foods15122046 (PMC13298098; doi:10.3390/foods15122046)
Supplement: Supplementary file 1 [file foods-15-02046-s001.zip › foods-4275474-supplementary.pdf]

Dough Functional Properties and Bread Quality of Stone-Milled Refined Flours in Comparison to Traditional HRS Wheat Flours

Deepa Pradhan [1,2], Amrita Ray [2] and Shahidul Islam [1] *

[1] Department of Plant Sciences, North Dakota State University, Fargo, ND 58108, USA
[2] Northern Crops Institute, Fargo, ND 58108-6050, USA
* Correspondence: Correspondence: shahidul.islam.1@ndsu.edu

__Supplementary Tables__:

Table S1. Glutopeak and RVA analyses of flour samples.

	Peak max. time	Torque max.	Aggregation energy	Peak viscosity	Hot paste viscosity	Final viscosity	Pasting temp.
**Varieties**							
Bolles	81.08^{b}	70.58^{a}	1810.09^{a}	2267.92^{a}	1724.25^{a}	2700.92^{a}	89.22^{a}
ND Frohberg	66.42^{c}	62.08^{b}	1652.39^{b}	2121.33^{ab}	1681.25^{ab}	2325.25^{b}	75.34^{b}
LCS Buster	96.25^{a}	53.58^{c}	1403.78^{c}	2003.75^{bc}	1623.50^{ab}	2415.00^{b}	86.28^{a}
Control	70.67^{bc}	53.00^{c}	1357.50^{c}	1885.83^{c}	1559.67^{b}	2371.08^{b}	85.50^{a}
**Milling method**					NS		NS
Stone mill	70.88^{b}	61.88^{a}	1583.67^{a}	1934.96^{b}	1616.92	2309.71^{b}	83.46
Roller mill	86.33^{a}	57.75^{b}	1528.21^{b}	2204.46^{a}	1677.42	2596.42^{a}	84.70
**Flour type**		NS					
WF	62.21^{b}	60.125	1480.62^{b}	2178.50^{a}	1707.13^{a}	2584.58^{a}	81.46^{b}
RF	95.00^{a}	59.50	1631.26^{a}	1960.92^{b}	1587.21^{b}	2321.54^{b}	86.71^{a}
**Variety*Mill**				NS	NS	NS	
ND Frohberg*Stone mill	72.33^{bc}	64.67^{bc}	1696.82^{ab}	2028.83	1641.83	2245.50	80.07^{c}
LCS Buster*Roller mill	111.33^{a}	53.00^{ef}	1416.68^{c}	2075.83	1655.67	2502.67	88.45^{ab}
Bolles*Roller mill	89.50^{b}	69.83^{ab}	1816.38^{a}	2434.33	1682.83	2848.67	89.98^{a}
ND Frohberg*Roller mill	60.50^{c}	59.50^{cd}	1607.95^{b}	2213.83	1720.67	2405.00	70.60^{d}
Bolles*Stone mill	72.67^{bc}	71.33^{a}	1803.79^{a}	2101.50	1765.67	2553.17	88.47^{ab}
LCS Buster*Stone mill	81.17^{b}	54.17^{def}	1390.88^{cd}	1931.67	1591.33	2327.33	84.10^{abc}
Control*Roller mill	84.00^{b}	48.67^{f}	1271.83^{d}	2093.83	1650.50	2629.33	89.78^{a}
Control*Stone mill	57.33^{c}	57.33^{de}	1443.18^{c}	1677.83	1468.83	2112.83	81.22^{bc}
**Variety*Flour**				NS			
LCS Buster* RF	121.33^{a}	50.83^{e}	1429.98^{b}	1880.50	1565.00^{ab}	2274.83^{cd}	87.49^{a}
Bolles* RF	98.67^{b}	67.00^{b}	1780.63^{a}	2214.33	1689.33^{a}	2626.17^{ab}	87.88^{a}
ND Frohberg* RF	73.83^{cd}	64.50^{bc}	1822.85^{a}	2071.00	1699.83^{a}	2264.17^{cd}	79.67^{b}
Control* RF	86.17^{bc}	55.67^{de}	1491.58^{b}	1677.83	1394.67^{b}	2121.00^{d}	91.78^{a}
ND Frohberg* WF	59.00^{d}	59.67^{cd}	1481.93^{b}	2171.67	1662.67^{a}	2386.33^{bcd}	71.01^{c}
LCS Buster* WF	71.17^{cd}	56.33^{de}	1377.58^{b}	2127.00	1682.00^{a}	2555.17^{abc}	85.06^{ab}
Bolles* WF	63.50^{d}	74.16^{a}	1839.55^{a}	2321.50	1759.17^{a}	2775.67^{a}	90.55^{a}
Control* WF	55.17^{d}	50.33^{e}	1223.42^{c}	2093.83	1724.67^{a}	2621.17^{ab}	79.22^{b}
**Variety*Mill*Flour**							NS
LCS Buster *Stone mill* RF	91.00^{bc}	53.33^{ef}	1456.15^{de}	1813.00^{c}	1592.33^{a}	2227.33^{bc}	86.37
Control *Roller mill* RF	117.33^{b}	48.67^{f}	1356.65^{ef}	2083.67^{abc}	1699.00^{a}	2613.67^{ab}	90.98
Bolles*Stone mill* RF	78.67^{cd}	71.33^{ab}	1852.42^{ab}	1946.67^{bc}	1682.33^{a}	2410.67^{abc}	89.42

Table S1. Glutopeak and RVA analyses of flour samples (continued).

	Peak max. time	Torque max.	Aggregation energy	Peak viscosity	Hot paste viscosity	Final viscosity	Pasting temp.
ND Frohberg*Stone mill* RF	80.00^{cd}	64.00^{bcd}	1791.59^{abc}	1869.33^{c}	1531.33^{a}	2079.33^{cd}	89.12
LCS Buster*Roller mill* RF	151.67^{a}	48.33^{f}	1403.82^{def}	1948.00^{bc}	1537.67^{a}	2322.33^{bc}	88.62
Bolles*Roller mill* RF	118.67^{b}	62.67^{bcde}	1708.83^{abc}	2482.00^{a}	1696.33^{a}	2841.67^{a}	86.35
ND Frohberg*Roller mill* RF	67.67^{cd}	65.00^{bc}	1854.10^{ab}	2272.67^{abc}	1868.33^{a}	2449.00^{abc}	70.22
ND Frohberg*Stone mill* WF	64.67^{cd}	65.33^{bc}	1602.05^{cd}	2188.33^{abc}	1752.33^{a}	2411.67^{abc}	71.03
LCS Buster*Roller mill* WF	71.00^{cd}	57.67^{cdef}	1429.55^{de}	2203.67^{abc}	1773.67^{a}	2683.00^{ab}	88.28
Bolles*Roller mill* WF	60.33^{cd}	77.00^{a}	1923.93^{a}	2386.67^{ab}	1669.33^{a}	2855.67^{a}	93.60
ND Frohberg*Roller mill* WF	53.33^{d}	54.00^{ef}	1361.80^{ef}	2155.00^{abc}	1573.00^{a}	2361.00^{abc}	70.98
Control*Stone mill* RF	55^{d}	62.67^{bcde}	1626.52^{bcd}	1272.00^{d}	1090.33^{b}	1628.33^{d}	92.58
Bolles*Stone mill* WF	66.67^{cd}	71.33^{ab}	1755.17^{abc}	2256.33^{abc}	1849.00^{a}	2695.67^{ab}	87.52
LCS Buster*Stone mill* WF	71.33^{cd}	55.00^{def}	1325.60^{ef}	2050.33^{abc}	1590.33^{a}	2427.33^{abc}	81.83
Control*Stone mill* WF	59.67^{cd}	52.00^{f}	1259.83^{ef}	2083.667^{abc}	1849.00^{a}	2597.33^{ab}	69.85
Control*Roller mill* WF	50.67^{d}	48.67^{f}	1187.00^{f}	2104.00^{abc}	1602.00^{a}	2645.00^{ab}	88.58

Note: Data represent mean. Values with different superscripts in a column differ significantly ($P < 0.05$).

WF: Whole-wheat flour, RF: Refined Flour, NS: non-significant.

Table S2. Farinograph analyses and baking quality of flour samples.

	WAC	DDT	Stability	MTI	Specific Volume(cc/g m)	Baking abs (14%)	Firmness
**Varieties**							
Bolles	73.06^{b}	10.35^{a}	33.29^{a}	11.33^{c}	5.51^{ab}	75.78^{a}	3692.75^{b}
ND Frohberg	73.88^{a}	8.79^{b}	13.81^{c}	16.08^{b}	5.70^{a}	76.38^{a}	2495.63^{b}
LCS Buster	66.73^{d}	8.39^{b}	19.43^{b}	14.92^{b}	4.26^{c}	70.22^{c}	7503.88^{a}
Control	169.68^{c}	5.78^{c}	11.40^{d}	21.83^{a}	5.25^{b}	74.61^{b}	2842.63^{b}
**Milling method**		NS					
Stone mill	71.69^{a}	8.52	17.73^{b}	17.00^{a}	4.94^{b}	75.16^{a}	3730.62^{b}
Roller mill	69.99^{b}	8.14	21.24^{a}	15.08^{b}	5.43^{a}	73.34^{b}	4536.81^{a}
**Flour type**				NS			
WF	74.22^{a}	8.07^{b}	16.75^{b}	16.75	4.23^{b}	78.74^{a}	5345.43^{a}
RF	67.45^{b}	8.59^{a}	22.21^{a}	15.33	6.13^{a}	69.74^{b}	2922.00^{b}
**Variety*Mill**					NS		NS
ND Frohberg*Stone mill	73.37^{b}	8.85^{bc}	14.96^{d}	15.83^{bc}	5.60	75.45^{b}	1977.75
LCS Buster*Roller mill	66.02^{e}	8.25^{c}	19.14^{c}	14.17^{bcd}	4.47	69.07^{d}	8208.25
Bolles*Roller mill	72.43^{c}	10.67^{a}	38.84^{a}	12.00^{cd}	5.84	75.28^{b}	3916.50
ND Frohberg*Roller mill	74.40^{a}	8.73^{bc}	12.65^{d}	16.33^{bc}	5.79	77.31^{a}	3013.50
Bolles*Stone mill	73.68^{ab}	10.03^{ab}	27.75^{b}	10.67^{d}	5.18	76.26^{ab}	3469.00
LCS Buster*Stone mill	67.45^{d}	8.54^{bc}	19.73^{c}	15.67^{bc}	4.05	71.36^{c}	6799.50
Control*Roller mill	67.10^{d}	4.90^{e}	14.34^{d}	17.83^{b}	5.59	71.66^{c}	3009.00
Control*Stone mill	72.25^{c}	6.65^{d}	8.47^{e}	17.83^{a}	4.90	77.55^{a}	2676.25
**Variety*Flour**		NS	NS	NS	NS		
LCS Buster* RF	62.38^{f}	8.73	22.27	13.00	5.10	65.41^{e}	5038.50^{b}
Bolles* RF	69.68^{d}	10.56	37.02	11.17	6.57	70.88^{d}	2435.25^{c}
ND Frohberg* RF	71.05^{c}	8.98	15.26	16.00	6.71	72.95^{c}	1609.75^{c}
Control* RF	66.70^{e}	6.08	14.30	21.17	6.12	69.75^{d}	2604.50^{c}
ND Frohberg* WF	76.72^{a}	8.60	12.36	16.17	4.68	79.81^{a}	3381.50^{bc}
LCS Buster* WF	71.08^{c}	8.05	16.60	16.83	3.42	75.02^{b}	9969.25^{a}
Bolles* WF	76.43^{a}	10.14	29.57	11.50	4.45	80.67^{a}	4950.25^{b}
Control* WF	72.65^{b}	5.48	8.50	22.50	4.37	79.46^{a}	3080.75^{bc}
**Variety*Mill*Flour**					NS		
LCS Buster *Stone mill* RF	63.33^{f}	9.93^{abc}	25.93^{c}	10.67^{cde}	4.44	66.20^{h}	6887.50^{b}
Control *Roller mill* RF	62.27^{fg}	4.53^{f}	20.15cd^{e}	12.00^{de}	6.65	64.51^{h}	2430.50^{cd}
Bolles*Stone mill* RF	70.77^{cd}	10.68^{ab}	32.36^{b}	9.33^{e}	5.80	71.07^{g}	3176.50^{bcd}

Table S2. Farinograph analyses and baking quality of flour samples (continued).

	WAC	DDT	Stability	MTI	Specific Volume(cc/gm)	Baking abs (14%)	Firmness
ND Frohberg*Stone mill* RF	70.47^{d}	8.79^{abcd}	16.13^{efg}	16.33^{bcde}	6.21	72.35^{fg}	1945.00^{d}
LCS Buster*Roller mill* RF	61.43^{g}	7.53^{cde}	18.61^{def}	15.33^{cde}	5.75	64.61^{h}	3189.50^{bcd}
Bolles*Roller mill* RF	68.60^{e}	10.45^{ab}	41.69^{a}	13.00^{de}	7.35	70.69^{g}	1694.00^{d}
ND Frohberg*Roller mill* RF	71.63c^{d}	9.17^{abcd}	14.38^{efgh}	15.67^{cde}	7.22	73.55^{ef}	1274.50^{d}
ND Frohberg*Stone mill* WF	76.27^{a}	8.91^{abcd}	13.78^{fgh}	15.33^{cde}	4.99	78.56^{bc}	2010.50^{d}
LCS Buster*Roller mill* WF	70.60^{cd}	8.96^{abcd}	19.67^{def}	13.00^{de}	3.18	73.52^{ef}	13227.00^{a}
Bolles*Roller mill* WF	76.27^{a}	10.89^{a}	35.99^{ab}	11.00^{de}	4.33	79.88^{ab}	6139.00^{bc}
ND Frohberg*Roller mill*WWF	77.17^{a}	8.29^{bcd}	10.93^{gh}	17.00^{bcd}	4.37	81.07^{a}	4752.50^{bcd}
Control*Stone mill* RF	71.13^{cd}	7.62^{cde}	8.46^{h}	30.33^{a}	5.59	74.99^{de}	2778.50^{cd}
Bolles*Stone mill* WF	76.60^{a}	9.38^{abcd}	21.13^{cd}	12.00^{de}	4.56	81.46^{a}	3761.50^{bcd}
LCS Buster*Stone mill* WF	71.57^{cd}	7.14^{de}	13.52^{fgh}	20.67^{bc}	3.65	76.53^{cd}	6711.50^{b}
Control*Stone mill* WF	73.37^{b}	5.68^{ef}	8.48^{h}	21.33^{bc}	4.21	80.11^{ab}	2574.00^{cd}
Control*Roller mill* WF	71.93^{c}	5.27^{ef}	8.52^{h}	23.67^{ab}	4.54	78.82^{b}	3587.50^{bcd}

Note: Data represent mean. Values with different superscripts in a column differ significantly ($P < 0.05$).

WF: Whole-wheat flour, RF: Refined Flour, NS: non-significant.

Table S3. Visual Inspection of bread samples.

	Symmetry	Crust Color	Crumb Color	Grain Texture
**Variety**		NS		
Bolles	7.38^{a}	6.88^{a}	4.50^{b}	5.62^{ab}
ND Frohberg	7.25^{a}	7.38^{a}	5.25^{a}	5.75^{a}
LCS Buster	6.00^{b}	5.75^{b}	4.38^{b}	4.38^{b}
Control	6.25^{b}	7.13^{a}	4.75^{b}	5.75^{a}
**Milling method**		NS		
Stone mill	6.50^{b}	6.87	4.06^{b}	4.93^{b}
Roller mill	6.93^{a}	6.68	5.37^{a}	5.81^{a}
**Flour type**				
WWF	5.93^{b}	6.00^{b}	2.93^{b}	4.37^{b}
Refined	7.50^{a}	7.56^{a}	6.50^{a}	6.37^{a}
**Variety*Mill**	NS		NS	NS
ND Frohberg*Stone mill	7.00	7.00^{ab}	4.50	5.25
LCS Buster*Roller mill	6.25	5.50^{d}	5.25	4.75
Bolles*Roller mill	7.50	6.75^{bc}	5.00	6.00
ND Frohberg*Roller mill	7.50	7.75^{a}	6.00	6.25
Bolles*Stone mill	7.25	7.00^{ab}	4.00	5.25
LCS Buster*Stone mill	5.75	6.00^{cd}	2.50	4.00
Control*Roller mill	6.50	6.75^{bc}	5.25	6.25
Control*Stone mill	6.00	7.50^{ab}	4.25	5.25
**Variety*Flour**		NS		NS
LCS Buster*Refined	6.25^{bc}	6.50	5.75^{c}	5.00
Bolles*Refined	8.25^{a}	7.75	6.50^{b}	7.00
ND Frohberg*Refined	8.25^{a}	8.25	7.50^{a}	7.00
Control*Refined	7.25^{ab}	7.75	6.25^{bc}	6.50
ND Frohberg*WWF	6.25^{bc}	6.50	3.00^{de}	4.50
LCS Buster*WWF	5.75^{c}	5.00	3.00^{de}	3.75
Bolles*WWF	6.50^{bc}	6.00	2.50^{e}	4.25
Control*WWF	5.25^{c}	6.50	3.25^{d}	5.00

Table S3. Visual Inspection of bread samples (continued).

	Symmetry	Crust Color	Crumb Color	Grain Texture
**Variety*Mill*Flour**	NS	NS	NS	NS
LCS Buster *Stone mill*Refined	5.50	7.00	4.00	4.00
Control *Roller mill*Refined	7.50	7.00	7.50	7.50
Bolles*Stone mill*Refined	8.00	8.00	5.00	6.00
ND Frohberg*Stone mill*Refined	7.50	8.00	6.00	5.00
LCS Buster*Roller mill*Refined	7.00	6.00	7.50	6.00
Bolles*Roller mill*Refined	8.50	7.50	8.00	8.00
ND Frohberg*Roller mill*Refined	9.00	8.50	9.00	9.00
ND Frohberg*Stone mill*WWF	6.50	6.00	3.00	5.50
LCS Buster*Roller mill*WWF	5.50	5.00	3.00	3.50
Bolles*Roller mill*WWF	6.50	6.00	2.00	4.00
ND Frohberg*Roller mill*WWF	6.00	7.00	3.00	3.50
Control*Stone mill*Refined	7.00	8.50	5.00	5.50
Bolles*Stone mill*WWF	6.50	6.00	3.00	4.50
LCS Buster*Stone mill*WWF	6.00	5.00	3.00	4.00
Control*Stone mill*WWF	5.00	6.50	3.50	5.00
Control*Roller mill*WWF	5.50	6.50	3.00	5.00

Note: Data represent mean. Values with different superscripts in a column differ significantly ($P < 0.05$).

WF: Whole-wheat flour, RF: Refined Flour, NS: non-significant.

Table S4. C-Cell analysis of bread samples.

	Brightness	Crumb Color			Number of cells	Cell volume (mm^3)
		L*	a*	b*		
**Variety**		NS	NS	NS	NS	
Bolles	76.19^{b}	36.35	2.82	16.79	3115.75	34.75^{ab}
ND Frohberg	76.24^{b}	35.63	3.00	15.14	2963.50	42.38^{a}
LCS Buster	80.38^{ab}	37.14	2.59	18.21	2850.63	27.0^{b}
Control	83.81^{a}	36.94	3.33	17.68	2935.50	32.63^{b}
**Milling method**				NS		NS
Stone mill	71.58^{b}	29.16^{b}	4.88^{a}	15.38	2895.63^{b}	33.75
Roller mill	86.73^{a}	36.26^{a}	2.61^{b}	16.78	3037.06^{a}	34.63
**Flour type**						**NS**
WF	62.27^{b}	24.54^{b}	5.85^{a}	16.04	2758.19^{b}	36.25
RF	96.04^{a}	40.88^{a}	1.64^{b}	16.12	3174.50^{a}	32.13
**Variety*Mill**		NS	NS		NS	
ND Frohberg*Stone mill	72.30^{de}	28.61	4.55	15.83^{bc}	2903.75	36.50^{ab}
LCS Buster*Roller mill	85.37^{abc}	34.47	2.73	14.17^{bcd}	2849.25	28.75^{b}
Bolles*Roller mill	87.92^{ab}	35.18	2.05	12.00^{cd}	3246.00	30.75^{b}
ND Frohberg*Roller mill	80.19^{bcd}	35.42	2.69	16.33^{bc}	3023.25	48.25^{a}
Bolles*Stone mill	64.46^{e}	26.82	4.72	13.51	2985.50	38.75^{ab}
LCS Buster*Stone mill	75.38^{cde}	28.65	4.96	15.88	2852.00	25.25^{b}
Control*Roller mill	93.45^{a}	39.98	2.97	19.57	3029.75	30.75^{b}
Control*Stone mill	74.17^{cde}	32.56	5.28	18.67	2841.25	34.50^{ab}
**Variety*Flour**		NS	NS	NS	NS	NS
LCS Buster* RF	96.17^{a}	39.35	1.65	15.16	3034.75	27.50
Bolles* RF	97.62^{a}	39.14	1.40	14.63	3310.00	32.00
ND Frohberg* RF	94.69^{a}	40.79	1.71	14.45	3229.00	35.50
Control* RF	95.68^{a}	44.23	1.79	20.24	3124.25	33.50
ND Frohberg* WF	57.80^{c}	23.24	5.53	14.33	2698.00	49.25
LCS Buster* WF	64.59^{bc}	23.76	6.03	16.45	2666.50	26.50
Bolles* WF	54.77^{c}	22.86	5.37	15.39	2921.50	37.50
Control* WF	71.94^{b}	28.31	6.46	17.99	2746.75	31.75

Table S4. C-Cell analysis of bread samples (continued).

	Brightness	Crumb color			Number of cells	Cell volume (mm^3)
		L*	a*	b*		
**Variety*Mill*Flour**	NS	NS	NS	NS	NS	NS
LCS Buster *Stone mill*Refined	83.27	33.81	3.59	15.17	2984.50	24.50
Control *Roller mill*Refined	115.30	52.19	-0.67	21.53	3267.50	30.50
Bolles*Stone mill*Refined	76.32	30.29	3.50	11.27	3100.0	34.0
ND Frohberg*Stone mill*Refined	76.61	33.72	3.43	12.76	3000.0	36.5
LCS Buster*Roller mill*Refined	109.06	44.89	-0.27	15.16	3085.0	30.5
Bolles*Roller mill*Refined	118.92	48.00	-0.69	17.99	3520.0	30.0
ND Frohberg*Roller mill*Refined	112.77	47.86	0.00	16.15	3458.0	34.5
ND Frohberg*Stone mill*WWF	67.99	23.51	5.67	14.18	2807.50	36.5
LCS Buster*Roller mill*WWF	61.68	24.04	5.74	16.30	2613.50	27.0
Bolles*Roller mill*WWF	56.93	22.36	4.80	15.03	2972.0	31.5
ND Frohberg*Roller mill*WWF	47.61	22.97	5.39	14.49	2588.50	62.0
Control*Stone mill*Refined	76.05	36.27	4.25	18.96	2981.0	36.5
Bolles*Stone mill*WWF	52.61	23.36	5.94	15.74	2871.0	43.5
LCS Buster*Stone mill*WWF	67.50	23.48	6.33	16.60	2719.5	26.0
Control*Stone mill*WWF	72.28	28.85	6.31	18.38	2701.50	32.5
Control*Roller mill*WWF	71.60	27.77	6.61	17.60	2792.0	31.0

Note: Data represent mean. Values with different superscripts in a column differ significantly ($P < 0.05$).

WF: Whole-wheat flour, RF: Refined Flour, NS: non-significant.

Supplementary Figures:

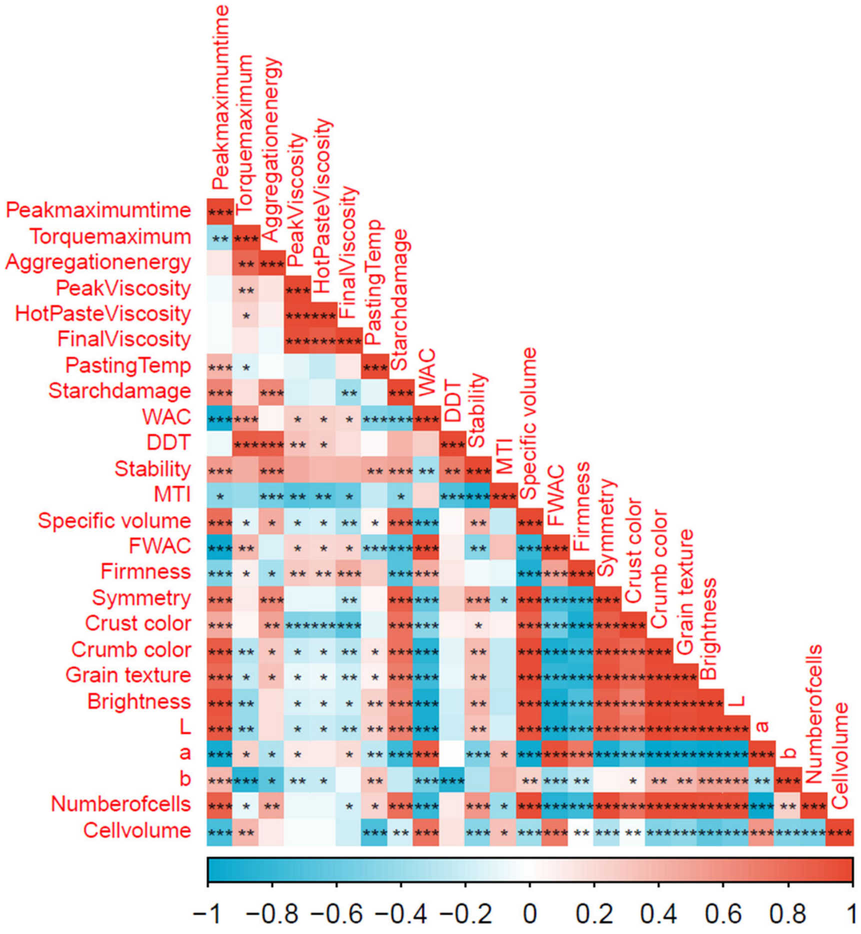

Figure S1. Pearson correlation matrix for functional, rheological, and bread quality parameters in wheat flour systems. Significance levels are indicated as $p < 0.05$ (*), $p < 0.01$ (**), and $p < 0.001$ (***).

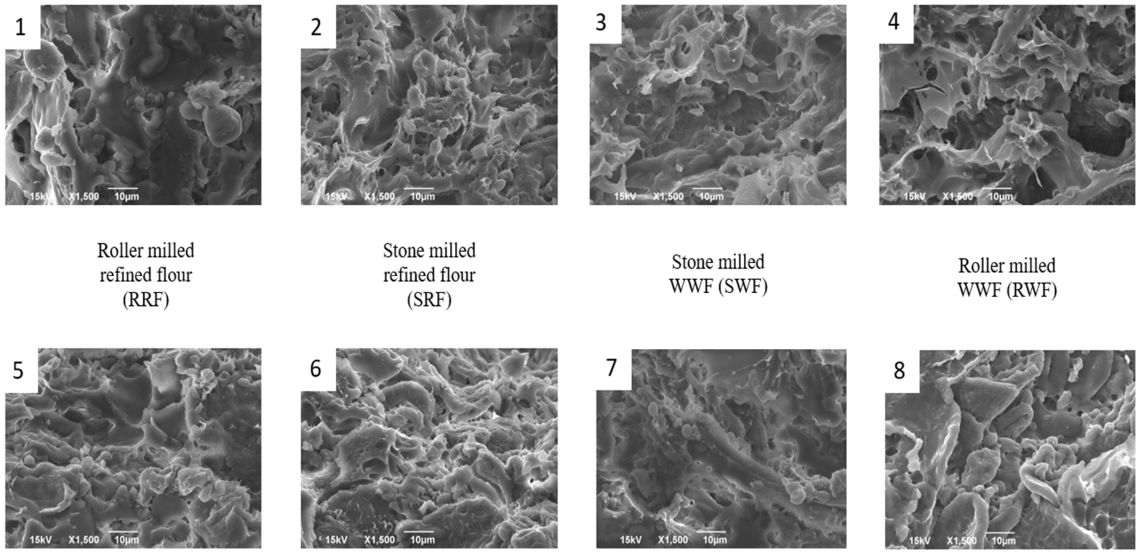

Figure S2. Scanning Electron Microscopy (SEM) images of bread samples of ND Frohberg (Top 4) and LCS Buster (Bottom 4) variety with 15kV×1500 magnification.
